# MA-EVIO: A Motion-Aware Approach to Event-Based Visual–Inertial Odometry

**DOI:** 10.3390/s25237381

**Published:** 2025-12-04

**Authors:** Mohsen Shahraki, Ahmed Elamin, Ahmed El-Rabbany

**Affiliations:** Department of Civil Engineering, Faculty of Engineering and Architectural Science, Toronto Metropolitan University, Toronto, ON M5B 2K3, Canada; agelamin@torontomu.ca

**Keywords:** sensor fusion, event-based visual-inertial odometry, motion awareness system, hybrid 6-DoF tracking, real-time indoor positioning

## Abstract

Indoor localization remains a challenging task due to the unavailability of reliable global navigation satellite system (GNSS) signals in most indoor environments. One way to overcome this challenge is through visual–inertial odometry (VIO), which enables real-time pose estimation by fusing camera and inertial measurements. However, VIO suffers from performance degradation under high-speed motion and in poorly lit environments. In such scenarios, motion blur, sensor noise, and low temporal resolution reduce the accuracy and robustness of the estimated trajectory. To address these limitations, we propose a motion-aware event-based VIO (MA-EVIO) system that adaptively fuses asynchronous event data, frame-based imagery, and inertial measurements for robust and accurate pose estimation. MA-EVIO employs a hybrid tracking strategy combining sparse feature matching and direct photometric alignment. A key innovation is its motion-aware keyframe selection, which dynamically adjusts tracking parameters based on real-time motion classification and feature quality. This motion awareness also enables adaptive sensor fusion: during fast motion, the system prioritizes event data, while under slow or stable motion, it relies more on RGB frames and feature-based tracking. Experimental results on the DAVIS240c and VECtor benchmarks demonstrate that MA-EVIO outperforms state-of-the-art methods, achieving a lower mean position error (MPE) of 0.19 on DAVIS240c compared to 0.21 (EVI-SAM) and 0.24 (PL-EVIO), and superior performance on VECtor with MPE/mean rotation error (MRE) of 1.19%/1.28 deg/m versus 1.27%/1.42 deg/m (EVI-SAM) and 1.93%/1.56 deg/m (PL-EVIO). These results validate the effectiveness of MA-EVIO in challenging dynamic indoor environments.

## 1. Introduction

Accurate positioning in indoor environments is critical for the reliable operation of autonomous systems, such as unmanned aerial vehicles (UAVs) and ground-based mobile robots. While global navigation satellite systems (GNSSs) can offer precise positioning in open outdoor settings, their performance is significantly degraded in indoor environments due to signal blockage, multipath effects, and structural attenuation [1]. Consequently, there is an increasing need for localization methods that ensure high precision and robustness in GNSS-denied environments, particularly in complex indoor spaces [2].

Given these limitations, researchers have turned to alternative indoor localization methods, with visual odometry (VO) and visual–inertial odometry (VIO) standing out as promising solutions [3]. By fusing data from cameras and inertial measurement units (IMUs), VIO enables real-time motion estimation for autonomous systems operating in GNSS-denied environments [4]. Although effective across a range of conditions, conventional frame-based VIO—which relies on standard image sequences—faces critical limitations in high-speed, low-light, or high-dynamic scenarios. Challenges such as motion blur, limited frame rates, and poor illumination often degrade image quality, resulting in significant tracking errors [5].

To overcome these issues, researchers have turned to event cameras, also known as dynamic vision sensors (DVS), which offer a compelling alternative to traditional frame-based cameras. These bio-inspired, event-driven sensors detect per-pixel brightness changes with microsecond latency, producing a continuous and asynchronous stream of sparse data. Unlike conventional cameras that capture full frames at fixed intervals, event cameras are highly effective under fast motion, sudden lighting changes, and high-dynamic-range conditions. Their inherent advantages—high temporal resolution, wide dynamic range (~140 dB), and low power consumption—make them particularly well-suited for real-time robotic perception and motion estimation tasks [5,6,7].

Despite these advantages, integrating event cameras into VIO pipelines poses substantial challenges. Frame-based VO/VIO algorithms process dense 2D intensity images, whereas event cameras emit sparse, asynchronous streams of brightness changes. This fundamental difference leads to issues, such as noisy measurements, sparse features in low-motion or low-texture scenes, and the need for entirely new approaches. As well, the lack of absolute intensity information and the edge-driven nature of event data often require specialized processing techniques for robust motion estimation [8,9].

To overcome these challenges and to fully exploit the unique characteristics of event data, researchers have developed a variety of specialized pose estimation algorithms tailored to the event-driven paradigm. These methods can be broadly categorized into feature-based, direct, and deep learning-based approaches, each offering distinct advantages depending on motion dynamics, scene texture, and computational constraints.

Recent deep learning-based methods have been proposed to exploit the rich spatiotemporal structure of event streams. DEVO [10] demonstrates end-to-end learning of camera motion directly from event data, while DEIO [11] further integrates IMU measurements to achieve metric 6-DoF motion estimation using a deep event–inertial pipeline. In parallel, [12] shows that sparse event streams can be transformed into structured voxel-graph representations for effective neural network processing, enabling learning on raw asynchronous data. Moreover, DH-PTAM [13], a deep hybrid stereo event–frame tracking and mapping system, combines learned features from both frame and event sensors to improve robustness in SLAM tasks. Despite their performance advantages, these deep learning approaches typically rely on labeled datasets and substantial computational resources, which limits their suitability for real-time deployment on resource-constrained robotic platforms and may reduce generalization in unseen environments.

Feature-based approaches focus on detecting repeatable structures, such as corners, edges, or geometric primitives, from the event stream and estimating motion by minimizing reprojection errors. Notable examples include Ultimate-SLAM (U-SLAM) [14] and ESVIO [15], which improve localization accuracy by leveraging hybrid features extracted from both event-based and frame-based data, in combination with inertial measurements from an IMU sensor. Some methods, such as IDOL [16] and PL-EVIO [17], further improve localization performance by incorporating higher-order features, such as lines. Other notable research includes EKLT-VIO [18], which employs Kalman filtering for asynchronous corner tracking. HASTE [19] also combines Harris corner detection [20] with event-based tracking to improve robustness under challenging conditions. Together, these feature-based approaches offer geometrically interpretable motion estimates and are often more resilient to drift, but they typically require careful event-to-feature association and can struggle in extremely fast or low-texture scenarios.

In contrast to feature-based approaches, direct methods estimate motion by minimizing photometric or temporal alignment errors on continuous event-based representations such as time surfaces, event frames, or gradient maps. Leveraging the high temporal resolution and dynamic range of event cameras, these methods are particularly effective in high-speed scenarios. For example, EVO [21] uses 2D–3D geometric alignment with a dense map from EMVS [22], while ESVO [23] performs edge-based 2D–3D registration on time surfaces using stereo input. EDS [24] minimizes photometric errors between events and image gradients for a full 6 degrees of freedom (6-DoF) of monocular tracking. Some approaches further rely on prebuilt 3D maps or constrain motion to pure rotation for simplicity [25]. More recently, ESVO2 [26] extends ESVO [23] by integrating IMU pre-integration in a tightly coupled visual–inertial pipeline, employing adaptive event accumulation for efficient contour sampling and combining static and temporal stereo for enhanced depth estimation. However, direct methods remain sensitive to event sparsity, map inconsistency, and noisy gradients, particularly in low-motion or challenging lighting conditions.

While feature-based methods can offer geometrically constrained optimization and direct methods can provide low-latency, high-frequency tracking, both face limitations under varying motion, lighting conditions, or sensor noise. Feature-based approaches can fail during high-speed motion when features are unreliable, while direct methods degrade in low-motion or low-event-rate scenarios. To address these issues, hybrid frameworks have emerged that seek to combine the complementary strengths of both paradigms [27]. However, many existing systems rely on static fusion strategies that fail to adapt to changing motion dynamics. This limitation motivates the development of motion-aware hybrid tracking systems, which dynamically adjust their tracking mode and sensor reliance—e.g., emphasizing event data during rapid motion and favoring image-based cues in low-speed or texture-rich scenes.

In this work, we propose a motion-aware event-based VIO (MA-EVIO), a hybrid system that dynamically integrates feature-based and direct tracking methods in an adaptive, motion-aware framework. Our main contributions are as follows:

- A novel motion-aware hybrid tracking framework that fuses direct and feature-based constraints, adjusting their contributions based on the robot’s motion.
- A motion-adaptive keyframe selection strategy that adjusts disparity and feature count thresholds using real-time motion classification and track consistency.
- An adaptive sensor fusion mechanism that prioritizes event data under aggressive motion and RGB frames in static scenes or slow motion, improving robustness across scenarios.
- Experimental validation on two public datasets [28,29].

This paper is organized as follows: Section 2 introduces the methodology proposed in this study. Section 3 details the datasets employed for evaluation. Section 4 presents the experimental results, while Section 5 offers a comprehensive analysis of the outcomes. Finally, Section 6 concludes the work, summarizing key findings and suggesting directions for future research.

## 2. Proposed Approach

**Figure 1:**
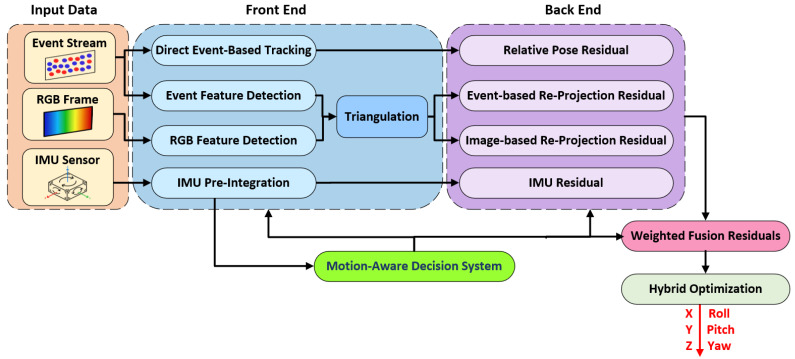
Overview of the proposed model architecture.

In this work, we propose MA-EVIO, a real-time system for estimating a full 6-DoF of motion—including position (x, y, z) and orientation (roll, pitch, yaw)—by fusing complementary sensing modalities from monocular events and RGB cameras with inertial measurements of linear acceleration and angular velocity. An overview of the MA-EVIO pipeline is presented in Figure 1.

At the core of MA-EVIO lies a motion-aware hybrid framework that adaptively combines feature-based and direct tracking methods depending on the current motion characteristics of the robot. During low-speed or texture-rich scenes, the system prioritizes feature-based tracking for stability and precision. In contrast, under fast motion or in low-texture environments, it shifts to direct methods that better exploit the high temporal resolution of event data. This adaptive mechanism allows MA-EVIO to maintain high pose accuracy and robustness across a wide range of dynamic conditions.

For sensor fusion, MA-EVIO employs a sliding-window, graph-based optimization backend that jointly minimizes multiple residuals within a unified framework. These include event-based geometric errors, such as reprojection residuals from tracked features, event-based photometric errors derived from the alignment event frames, image-based geometric constraints from standard frames, and IMU pre-integration terms that capture inertial motion dynamics. In the following subsections, we provide a detailed description of how MA-EVIO tightly couples these heterogeneous inputs within its motion-aware optimization framework.

### 2.1. Initialization

Monocular VIO systems require an explicit and carefully designed initialization phase to estimate key quantities such as metric scale, gravity direction, sensor biases, and velocities. Unlike stereo systems, monocular VIO suffers from scale ambiguity. Hence, the fusion of visual estimates with IMU measurements is essential to achieve a globally consistent metric reconstruction of the trajectory. We follow a standard pipeline [30] that fuses visual structure from motion (SfM) [31] with IMU data to recover metric scale, gravity direction, and sensor biases.

Initialization begins by selecting two keyframes with sufficient parallax. Sparse features are tracked, and the five-point algorithm [32] estimates their relative pose up to a scale. Triangulated 3D points define an initial sparse map. Subsequent camera poses are recovered using Perspective-n-Point [33], followed by a global bundle adjustment [34] to refine both structure and motion. At this point, the trajectory is accurate but unscaled and not aligned to the direction of gravity. To produce a consistent and physically meaningful trajectory, the up-to-scale visual structure obtained from monocular SfM must be aligned with the inertial data. Assuming that the extrinsic calibration between the camera and body (IMU) frames is known—specifically, the rotation $R_{c}^{b}$ and translation $T_{c}^{b}$ from the camera to the body frame—the goal is to align or reconcile the visual trajectory obtained from the camera with the motion measurements provided by the IMU.

First, gyroscope data is integrated between consecutive frames to compute relative rotations in the body (IMU) frame. These IMU-based rotations are then used for short-term motion prediction. By exploiting the much higher sampling rate of IMU sensor compared to the camera, these relative rotations help stabilize orientation and synchronize the inertial and visual data streams. With the rotational alignment established, the next step is to recover the direction of gravity and the metric scale, both of which are missing from the monocular visual trajectory. To do this, we estimate a rotation matrix $R_{v}^{g}$ that aligns the SfM visual frame with a gravity-aligned global map, which serves as a unified reference frame for localization. The transformation of the camera poses from the SfM visual frame ($R_{c_{i}}^{g},T_{c_{i}}^{g}$) to the gravity-aligned global frame is as follows:

(1) $$R_{c_{i}}^{g}=R_{v}^{g}.R_{c_{i}}^{v},T_{ci}^{g}=s.R_{v}^{g}.T_{c_{i}}^{v},$$

To compute the metric scale s, we leverage the consistency between visual displacement and IMU-estimated displacement over a short temporal window. Specifically, for consecutive frames i and i+1, the scale is estimated as follows:

(2) $$s=\frac{{\left\|∆p_{i,i+1}^{IMU}\right\|}_{2}}{{\left\|∆p_{i,i+1}^{V}\right\|}_{2}},$$

where $∆p_{i,i+1}^{V}=T_{c_{i}+1}^{V}-T_{c_{i}}^{V}$ is the relative translation obtained from the visual SfM trajectory (up to scale), and $∆p_{i,i+1}^{IMU}$ is the corresponding relative displacement obtained by double integrating IMU linear acceleration in the body frame while compensating for gravity and bias. The operator ${\left\|.\right\|}_{2}$ denotes the Euclidean norm. To improve robustness against noise, the final scale factor s is computed as the median value over a window of N consecutive frame pairs:

(3) $$s={median}_{i\in [1,N]}\left(\frac{{\left\|∆p_{i,i+1}^{IMU}\right\|}_{2}}{{\left\|∆p_{i,i+1}^{V}\right\|}_{2}}\right),$$

Physically, this scale factor enforces metric consistency between the visual trajectory (which is scale-ambiguous due to monocular vision) and the inertial motion estimates, thus converting the trajectory into real-world metric units and aligning it with the direction of gravity.

Once the camera pose has been aligned to the gravity-consistent global frame, the corresponding pose of the IMU frame is recovered using the known rigid-body transformation between the camera and IMU. Specifically, since $R_{c}^{b}$ maps camera coordinates to body coordinates, the IMU body rotation and translation in the global frame are computed as follows:

(4) $$R_{b_{i}}^{g}=R_{c_{i}}^{g}.{\left(R_{c}^{b}\right)}^{T},T_{b_{i}}^{g}=T_{c_{i}}^{g}+R_{c_{i}}^{g}.T_{b}^{c},$$

where ${\left(R_{c}^{b}\right)}^{T}$ is inverse of the orthogonal matrix $R_{c}^{b}$, and $T_{b}^{c}$ is the IMU origin in the camera frame. This alignment allows for the joint estimation of gravity and scale by minimizing the residuals between visual motion and inertial predictions within a temporal sliding window that maintains recent keyframes and IMU measurements for optimization. With the trajectory aligned and scaled, we estimate the full set of motion parameters required for initialization. These include the velocity $v_{i}$ at each frame, the accelerometer bias $b_{a}$, the refined gravity direction, and the confirmed metric scale s. In addition, the gyroscope bias $b_{g}$ is estimated to correct for systematic drift in orientation, further improving integration accuracy. These parameters are solved by minimizing the residuals between IMU pre-integrated measurements and transformed visual estimates using a nonlinear least-squares optimization over the sliding window.

These initial estimates serve as the starting point for a tightly coupled visual–inertial estimator, which continuously refines the full system state during real-time operation. The entire initialization pipeline is designed to run automatically and robustly, without requiring prior knowledge of the system state or motion pattern. This makes the method suitable for deployment in challenging, real-world scenarios where reliable, on-the-fly initialization is essential.

### 2.2. Event Representations

Event cameras are relatively new sensors that detect changes in brightness at the pixel level and report them as a stream of asynchronous events, rather than capturing full-intensity images at fixed intervals. Each event is generated when the log intensity at a pixel exceeds a preset threshold, typically due to motion in the scene or movement of the camera itself. An individual event can be represented as e=(u,v,t,p), where (u,v) are pixel coordinates, t is the timestamp, and p indicates the polarity of the change—positive for increasing intensity and negative for decreasing. The generation of an event is governed by a threshold $T_{Threshold}$, such that an event occurs when the change in log intensity at a pixel satisfies the following:

(5) $$\left\{\begin{array}{cc} L\left(u,v,t+\Delta t\right)-L\left(u,v,t\right)\geq \mathrm{Tthreshold}, & \mathrm{for}\;p=+1,\\ L\left(u,v,t+\Delta t\right)-L\left(u,v,t\right)\leq -\mathrm{Tthreshold}, & \mathrm{for}\;p=-1, \end{array}\right.$$

where p can be −1 or +1 and it encodes the polarity of the brightness change. It is worth noting that Equation (5) is defined in the logarithmic intensity domain L=log(I), making the event generation process invariant to global illumination changes by depending on relative contrast rather than absolute brightness。

Due to the sparse and high-temporal-resolution nature of event data, individual events carry limited contextual information. As a result, it is common to aggregate events over a short temporal interval into more structured representations that are better suited for processing [9]. In our system, we make use of two such representations: the time surface (TS) and the event mat, each offering complementary strengths for event-based VIO.

The adaptive TS is a 2D map that encodes the spatiotemporal activity of recent events, where each pixel stores a value that decays exponentially with the time elapsed since its last event. This value may also incorporate the polarity of the event to preserve contrast information. Formally, for a given pixel location x=(u,v) and current time t, the adaptive TS is defined using an exponential decay function centered on the timestamp of the most recent event at that location:

(6) $$T\left(x,t\right)=p.exp\left(-\frac{t-t_{last}(x)}{\eta }\right),$$

where $t_{last}$ denotes the timestamp of the most recent event at pixel x, p is the event polarity, which can be either +1 or −1, and η is an adaptive parameter that controls the rate of temporal decay. In contrast to fixed-decay approaches, our method adopts an event-adaptive decay mechanism, in which the decay rate η is dynamically adjusted according to the event generation rate. This strategy addresses a key limitation of conventional TS formulations, where a fixed decay parameter cannot accommodate variations in camera motion dynamics. During low-speed motion, the event occurrence frequency decreases, leading to sparse and low-contrast TS images that degrade their usefulness for subsequent processing. Conversely, during fast motion, the high event rate may cause excessive temporal overlap if the decay is not properly controlled. To alleviate both issues, we use the event density within a temporal window as an indirect indicator of motion activity and continuously adapt the decay rate based on this measure. Specifically, when the event rate is low (corresponding to slow camera motion), the decay parameter is increased to enhance contrast and compensate for sparsity. When the event rate is high (corresponding to fast camera motion), a shorter decay is applied to preserve temporal consistency and prevent motion-induced smearing. This event-driven adaptive decay strategy ensures that the generated TS images remain informative across a wide range of motion speeds, thereby improving robustness in downstream tasks such as feature detection, event-based tracking, and visual–inertial state estimation. Further details on the event-adaptive time-surface formulation and implementation can be found in [15].

The event mat is a binary 2D representation generated by accumulating asynchronous events over a fixed temporal window Δt. Within this window, each pixel is assigned a value of 1 if any event occurred at that location; otherwise, it remains at zero. This process transforms the sparse event stream into a dense 2D image, defined as follows:

(7) $$E_{t_{0}}=\left\{e|t_{0}<t<t_{0}+\Delta t\right\},$$

where $E_{t_{0}}$ denotes the set of all events collected during the time window starting at $t_{0}$, with Δt representing the duration of the accumulation interval.

This representation is particularly valuable for tasks requiring spatial continuity, such as 2D–2D alignment and direct pose tracking. In our system, event mats are constructed at key time steps for use in hierarchical image alignment. For each pair of frames (i,k), we generate two event mats, $E_{i}$ (reference) and $E_{k}$ (current), and perform direct alignment by minimizing the photometric error between them. This alignment is based on the transformation that best warps the current image to match the reference, expressed through the following:

(8) $$E(\xi )=\sum_{x\in \Omega }{\left\|I_{k}\left(\pi \left(T\left(\xi \right).X\right)\right)-I_{i}\left(\pi \left(X\right)\right)\right\|}^{2},$$

where ξ represents the pose perturbation, $T\left(\xi \right)$) denotes SE(3) transformation (i.e., a rigid body motion in 3D space), π(⋅) is the projection model, and $I_{i}$ and $I_{k}$ are the reference and current event mat images, respectively. Alignment is performed using a coarse-to-fine strategy with image pyramids and iterative optimization producing not only the optimal pose but also the associated information matrix. To maintain geometric consistency and robustness across varying motion conditions, we assess the alignment quality using confidence metrics and selectively update the reference event mat when necessary. This enables the event mat to serve as a reliable visual constraint in the optimization backend, where pose residuals derived from the alignment are integrated as cost terms in the factor graph. By complementing the temporal sensitivity of the adaptive TS with the spatial structure of the event mat, our system achieves high-quality motion estimation, even in challenging scenes with rapid dynamics or sparse texture.

### 2.3. Hybrid Tracking Module

To achieve robust and accurate motion estimation, we design a hybrid tracking module that tightly integrates two complementary approaches: feature-based event visual–inertial odometry (EVIO) and event-based direct photometric alignment. These modules operate in parallel and contribute independent, complementary constraints to a unified sliding-window optimization backend. By combining the high spatial consistency of feature reprojection with the dense temporal fidelity of event photometric alignment, the system maintains performance across a wide range of motion dynamics, textures, and illumination conditions.

**Figure 2:**
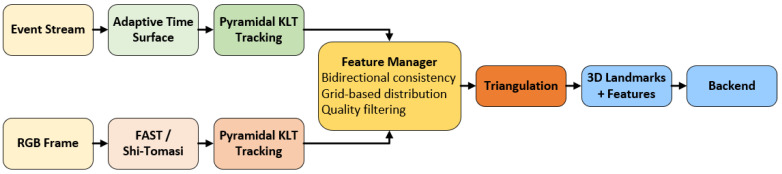
Overview of the proposed feature-based tracking module.

MA-EVIO’s feature-based tracking pipeline, depicted in Figure 2, implements a multi-modal, adaptive framework for robustly tracking corners using both standard RGB frames and asynchronous event streams. Unlike traditional approaches that rely solely on frame-based imagery, our system leverages the microsecond-level resolution of event cameras to preserve feature tracks under high-speed motion, motion blur, and low-light conditions. This fusion is particularly critical in aerial and mobile robotics scenarios where robustness and responsiveness are essential.

From the RGB camera, we extract salient corner features using a Shi–Tomasi detector [35] or, optionally, a FAST detector [36], depending on environmental conditions such as texture richness or motion blur. The selection between the two detectors is governed by a heuristic or learned policy based on frame-level quality metrics (e.g., number of successfully tracked features or image gradient variance). This switching logic is implemented in the feature manager module, which evaluates the current scene and dynamically chooses the appropriate detector. All features are subsequently tracked using pyramidal Kanade–Lucas–Tomasi (KLT) [37] optical flow. The pyramidal structure enables multi-scale matching, which is critical when relative motion causes significant disparity. Each feature is tracked across successive RGB frames using forward–backward flow consistency checks to reject unreliable correspondences. To ensure temporal consistency and spatial coverage, a grid-based feature management strategy is employed to maintain a uniform distribution of features. Features are undistorted using camera intrinsic and projected onto the normalized plane for further processing. If the number of key features drops below a predefined threshold, new features are detected and initialized. These RGB features contribute 2D–3D reprojection factors to the backend once triangulated.

Complementing the RGB tracking, our system performs event-based corner tracking on the adaptive time surface T(x,t), a continuously updated representation of recent event activity. The time surface is defined according to Equation (6) which emphasizes recent motion patterns while maintaining robustness under slow motion or scene stasis.

Corner features on the time surface are tracked using a pyramidal KLT tracker, with the initial optical flow seeded by IMU-predicted motion. This fusion ensures reliable tracking even under fast rotations or degraded event rates. Bidirectional flow consistency and residual thresholds are applied to validate tracks, discarding ambiguous or noisy candidates. As with the frame-based pipeline, if the number of surviving event corners falls below a minimum threshold, a FAST corner detector is applied directly to the time surface. Detected corners are then filtered through non-maximum suppression, corner strength ranking, and outlier rejection.

**Figure 3:**
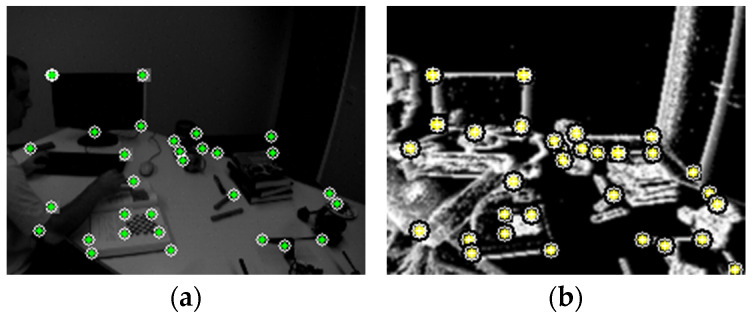
FAST feature detection on the DAVIS240c dataset [28]. (**a**) RGB frame. (**b**) Adaptive time surface.

Tracked features—whether from events or frames—are triangulated into 3D via inverse depth parameterization, which provides better numerical stability and supports tracking across large baselines. If a feature track maintains sufficient length and consistency, the triangulated 3D point is promoted to a landmark. Each landmark is associated with a corresponding observation in the local sliding window and contributes reprojection residuals to the backend graph optimization. This multi-source triangulation and promotion process ensures that landmarks are well-constrained and persistent across time. To maintain robustness across diverse motion conditions, the tracking module incorporates a motion classification module, which leverages visual disparity, event rate, and IMU angular velocity to infer motion patterns. Based on the classification, the tracker dynamically adjusts parameters such as KLT search radius, detection thresholds, and outlier rejection criteria. For example, under rapid motion, search windows are expanded, and stricter bidirectional consistency checks are relaxed. This dynamic adaptation enables robust feature maintenance even in aggressive maneuvers or texture-sparse environments. Figure 3 illustrates the feature detection process within our tracking pipeline. Subfigure (a) shows feature extraction on an RGB frame, while subfigure (b) demonstrates feature detection on the adaptive time surface generated from asynchronous events.

In parallel, the system performs event-based odometry using a direct alignment method known as event mat tracking [27]. This approach leverages the photometric consistency between temporally accumulated event representations to estimate relative motion through dense image alignment techniques. To estimate relative motion, the system maintains a reference event mat $E_{i}$ and aligns each new frame $E_{k}$ against it. The alignment task is formulated as a nonlinear least-squares optimization problem that minimizes the photometric error between warped pixel intensities from the reference and the current event mat. The optimization variable is the relative pose ${\Delta T}_{i\to k}\in SE(3)$, representing the rigid-body transformation from the reference frame i to the current frame k, where SE(3) denotes the Special Euclidean group of 3D rotations and translations. Using the Inverse Compositional Lucas–Kanade (IC-LK) method [38], the reference event mat is warped according to the current pose estimate, and residuals are computed as the differences between the warped reference pixels and those in the current frame.

The optimization proceeds through a hierarchical pyramid approach—starting from coarse image scales and moving to finer ones—to ensure robustness against large motions. At each pyramid level, the residuals are linearized using image gradients, and the Jacobian matrix of residuals with respect to the pose parameters and the corresponding Hessian matrix are computed. The update vector δξ∈SE(3) representing a small incremental motion, is then solved and applied to update the current pose estimate ${\Delta T}_{i\to k}\in SE(3)$ via the exponential map:

(9) $${\Delta T}_{i\to k}\leftarrow \exp\left(\delta \xi \right).{\Delta T}_{i\to k},$$

This iterative process continues until convergence of the nonlinear least-squares optimization—judged by the norm of the pose update $\left\|\delta \xi \right\|$—or until a fixed number of iterations is reached.

After alignment, the system computes an information matrix that quantifies the confidence in the resulting pose estimate. This confidence is crucial for integration into the backend graph optimizer. Furthermore, a quality assessment module evaluates the reliability of the alignment using metrics such as residual error and convergence rate. If the alignment is deemed reliable and a significant motion or time threshold has passed, the reference event mat is updated. Rather than replacing it completely, the system blends new observations with the existing mat to preserve temporal consistency while adapting to changing scene content. The final relative transformation ${\Delta T}_{i\to k}$ is estimated from event mat alignment, packaged as a pose–pose constraint, and passed to the MA-EVIO backend for optimization. This constraint expresses the error between the observed relative motion (from event alignment) and the current estimated poses of the reference and current frames in the global frame. The residual is defined as follows:

(10) $$r\left(z_{direct},\chi \right)={\left\|{\Delta T}_{i\to k}.T_{\omega }^{b_{i}^{-1}}.T_{\omega }^{b_{k}}\right\|}^{2},$$

where $T_{\omega }^{b_{i}^{-1}}\in SE(3)$ is the inverse of the estimated body pose at time i in the global frame. It transforms coordinates from the body frame at time i to the world frame. Similarly, $T_{\omega }^{b_{k}}\in SE(3)$ is the estimated pose of the body at time k in the global frame. The variable χ denotes the state vector in the EVIO graph, which includes all estimated poses and potentially other variables such as velocities and IMU biases. The function $r\left(z_{direct},\chi \right)$ represents the residual between the direct motion measurement $z_{direct}$ (obtained from event mat alignment) and the current state estimate χ, quantifying how well the system’s estimate aligns with the observed relative transformation.

**Figure 4:**
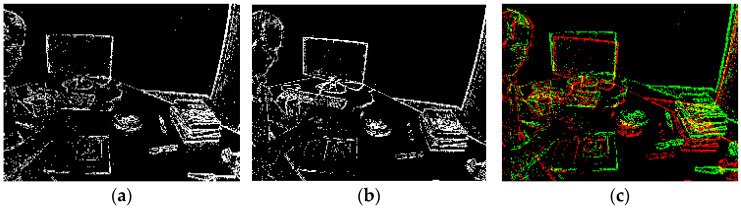
Sample of direct event-based alignment on DAVIS240c dataset [24]: (**a**,**b**) Two consecutive evet frame used for 2D–2D alignment. (**c**) Result of the alignment, where overlapping events from the current and previous event frames are visualized in green and red.

This cost is incorporated into the graph optimizer using the event mat pose error class, which computes the residuals and their Jacobian matrices with respect to the pose parameters of the estimated frames. The associated information matrix provides uncertainty weighting, and robust loss functions can be applied to suppress outliers. Finally, the system operates in a sliding window: as new frames arrive, the current event mat $E_{k}$ becomes the new reference $E_{i}$, and a new image is created from recent events. The cycle repeats, continuously producing high-confidence pose estimates. This architecture supports real-time operation and ensures that event-only tracking remains stable, accurate, and drift-aware. Figure 4 illustrates direct event-based alignment on the DAVIS240C dataset. Subfigure (a,b) show two consecutive event frames, constructed over short temporal windows, which serve as the reference and current frames for alignment, while (c) presents the result of the alignment, where overlapping events from the two event frames are visualized in green and red. The close correspondence between the two event mats highlights the effectiveness of the IC-LK method in accurately warping the current frame to the reference, leveraging dense photometric consistency from asynchronous event data for robust pose estimation under dynamic motion and illumination changes.

### 2.4. Motion Aware Module

Our motion-aware optimization framework employs a multi-stage classifier to evaluate and categorize motion characteristics and adjust residual weighting within the state estimation pipeline. The classifier follows a hierarchical decision strategy that integrates complementary inertial, visual, and event-based cues to estimate both motion intensity and sensor reliability in real time. Motion is categorized into four discrete intensity levels: static (near-zero motion), slow (gentle and controlled motion), moderate (nominal operational motion), and aggressive (rapid, high-dynamic motion). Classification is performed through multi-modal fusion, beginning with IMU measurements. The IMU-based classifier computes a weighted motion score (MS) defined as follows:

(11) $$MS=max(0.4\left|a\right|,0.3\left|\omega \right|)+0.2\left|v\right|+0.1\left|\alpha \right|,$$

where $\left|a\right|$ is the gravity-compensated linear acceleration magnitude (m/s^2^), $\left|\omega \right|$ is the angular velocity magnitude (rad/s), $\left|v\right|$ is the estimated linear velocity magnitude (m/s), and $\left|\alpha \right|$ is the angular acceleration magnitude (rad/s^2^). Gravity compensation is performed by rotating the raw accelerometer measurements into the global frame using the estimated IMU orientation and subtracting the gravity vector $g={\left[0,\;0,\;9.81\right]}^{T}$ (m/s^2^). Specifically, the linear acceleration is computed as follows:

(12) $$a_{lin}=R_{b}^{g}a_{raw}-g,$$

where $R_{b}^{g}$ is the rotation matrix from the body (IMU) frame to the global frame obtained from the IMU quaternion. The magnitude $\left|a\right|$ used in the motion score is then given by $\left|a\right|={\left\|a\right\|}_{2}$. The weighting coefficients (0.4,0.3,0.2,0.1) are determined through a combination of empirical evaluation and physical reasoning. Empirically, multiple coefficient configurations were evaluated using diverse datasets that include static scenes as well as slow, nominal, and aggressive motion with rapid rotations and accelerations. Each configuration was assessed according to its motion classification stability and its impact on downstream state estimation accuracy. Physically, larger weights are assigned to translational acceleration $\left|a\right|$ and angular velocity $\left|\omega \right|$ because they directly reflect instantaneous platform dynamics and have the strongest influence on visual and event-based sensing. High acceleration causes motion blur and degrades frame-based tracking, while high angular velocity introduces severe viewpoint changes and event bursts, both of which significantly affect feature reliability. In contrast, linear velocity $\left|v\right|$ reflects accumulated motion rather than instantaneous change, and angular acceleration $\left|\alpha \right|$ is more sensitive to noise due to numerical differentiation. Therefore, they are assigned lower weights to act as stabilizing secondary indicators rather than dominant factors. The use of the max operator ensures that either strong rotation or strong translation alone can sufficiently increase the motion score, reflecting realistic motion scenarios. Based on this score, the motion state is categorized into four regimes:

(13) $$MotionType=\left\{\begin{array}{cc} MS\leq 0.05 & \to Static\\ 0.05<MS\leq 0.5 & \to Slow\\ 0.5<MS\leq 2.5 & \to Moderate\\ MS>2.5 & \to Agressive \end{array}\right..$$

The threshold values are also selected experimentally, based on statistical analysis of motion score distributions over sequences with different motion dynamics (static, slow, moderate, and aggressive). Specifically, we analyzed some sequences in both datasets, including sequences with smooth motion, general navigation, and high-speed maneuvers. In addition, these thresholds were cross-validated using visual and event-based feature quality metrics, such as the number of tracked features, track longevity, and event consistency. We observed that when MS>2.5, both RGB and event-based feature tracking degraded significantly due to motion blur and extreme viewpoint change. Conversely, when MS≤0.05, nearly constant feature counts and negligible motion indicated quasi-static conditions dominated by sensor noise. The intermediate thresholds (0.05,0.5,2.5) were chosen to maximize inter-class separation while minimizing overlap between regimes and ensuring that each motion state corresponds to a distinct level of physical dynamics and sensor reliability.

To suppress rapid oscillations between motion modes, a mode stability filter is employed. This filter introduces hysteresis by requiring a motion state to be consistently classified over several consecutive frames before a transition is confirmed. A transition into the aggressive state—which indicates a significant increase in motion intensity from the static, slow, or moderate regimes—requires the most stringent validation of 7 consecutive frames. This prevents the system from prematurely entering a high-dynamics mode due to transient spikes. Conversely, a transition out of the aggressive state back to a calmer state (slow or moderate) is permitted after only 3 consecutive frames, allowing for quicker recovery when intense motion subsides. All other transitions between non-aggressive states are validated over a buffer of 5 consecutive frames.

Beyond IMU cues, the classifier also evaluates visual feature tracking quality using metrics such as feature inlier ratios and temporal persistence of tracked landmarks. These visual indicators are complemented by analysis of event streams, which provide robust motion cues in high-speed scenarios where conventional frame-based features degrade.

The output of the classification system directly controls the residual weighting strategy in the optimization backend. The tracking mode decision mechanism uses hybrid tracking rather than exclusive switching. Frame-based (RGB) tracking serves as the primary mode for feature extraction using Lucas–Kanade optical flow, while event-driven tracking operates in a supplementary mode using accumulated event frames (time surfaces). The system tags each feature with its source (RGB or EVENT), and feature weighting is applied during backend optimization based on both motion type and feature source. This linkage between the classified motion state and the backend optimization is concretely realized through a distinct residual weighting profile for each motion type.

In static conditions, the system capitalizes on highly stable and accurate visual features. Visual reprojection residuals are assigned a dominant weighting to ensure high spatial accuracy, while inertial residuals are heavily de-weighted to prevent state estimate corruption from noise-dominated measurements. Due to the lack of sufficient motion to generate a meaningful event stream, event-based features are typically disabled. As motion transitions into the slow regime, gentle movement introduces slight feature displacement. Visual tracking remains robust and thus retains its primary role, while inertial terms are cautiously introduced with low weights to provide essential high-frequency pose updates between frames, mitigating the influence of low-velocity IMU noise. Event-based features begin to appear but are often sparse and ambiguous; consequently, they are either ignored or assigned a minimal weight to avoid introducing noise.

Upon entering the moderate motion regime, noticeable movement introduces the first signs of visual degradation, such as slight motion blur. The framework shifts to a balanced sensor fusion strategy. The weighting of visual constraints is slightly reduced but remains crucial for absolute accuracy, whereas inertial constraints are significantly upweighted to provide smooth, high-bandwidth motion tracking. Event-based features, now becoming more consistent and plentiful, are formally incorporated as a supplementary data source, with their influence dynamically calibrated based on their measured consistency with the current motion estimate.

This adaptive balance is pushed further in the aggressive status, where rapid, high-dynamic motion presents the most significant sensor reliability challenges. To maintain robustness, the system executes a protective, multi-layered adaptation. The weights for visual residuals are substantially reduced to mitigate errors from severe motion blur, and IMU constraints are selectively filtered based on saturation detection. Critically, event-based feature tracking and Event MAT residuals are promoted to a primary data source. Their influence is dynamically boosted to compensate for the concurrent impairments in both the visual and inertial streams, thereby ensuring tracking continuity where conventional methods would fail. The transition of residual weights between these discrete motion states is smoothly interpolated based on the continuous motion score, ensuring stable and progressive adaptation.

This multi-modal adaptation is implemented through motion-aware covariance scaling that smoothly interpolates between motion types. Rather than employing discrete threshold-based switching, the system achieves continuous adaptation through smooth sigmoid-like interpolation functions that modulate both residual weights and the event time-surface decay parameter as a function of motion intensity. The interpolation is based on the classifier’s continuous confidence metrics and further enhanced by reliability checks applied to each sensor stream. The weight adaptation logic also incorporates quality-aware marginalization and adaptive loss tuning, which dynamically adjust robustness parameters according to sensor reliability, as further detailed in Section 2.5. This design guards against overreliance on any single modality and maintains consistent performance even under partial sensor degradation.

Underlying this entire framework is a principled approach to sensor fusion that explicitly maintains the relationship between motion classification and residual weighting through measurable, quantifiable criteria. Each adjustment in residual weighting can be directly traced to specific classifier outputs and the sensor measurements that informed them. The implementation avoids heuristic weighting rules in favor of this systematic approach, where all adaptations are grounded in observable characteristics of the sensor data and motion dynamics. This design philosophy enables the optimization framework to maintain consistent performance across the full spectrum of operating conditions, from completely static environments to highly dynamic motion scenarios.

### 2.5. Hybrid Optimization Technique

To solve for the system state under varying motion and sensor conditions, we adopt a hybrid optimization strategy grounded in a maximum a posteriori (MAP) estimation framework, implemented via nonlinear least squares using the Ceres Solver [39]. The nonlinear least-squares formulation is particularly suited for this problem, as it enables joint optimization of camera poses and other state variables by minimizing the residuals arising from inherently nonlinear measurement models [14]. This formulation tightly fuses asynchronous event data, traditional visual features, and inertial measurements into a unified probabilistic graph. The optimization integrates residuals from (1) IMU pre-integration, (2) visual reprojection errors, and (3) event-based photometric alignment, all maintained within a sliding window structure and refined through robust loss functions and marginalization. Residual weights are dynamically adjusted based on motion classification module output, enabling the system to prioritize the most reliable modalities in each regime. For instance, during aggressive motion, visual constraints are down-weighted due to motion blur, while event-based features and event mat—which remain reliable under high dynamics—are emphasized. This hybrid, motion-aware formulation enables robust and precise state estimation across diverse motion scenarios. At each time step t, the estimated system state is represented as follows:

(14) $$\chi =[p_{b}^{g},q_{b}^{g},v_{b}^{g}],$$

where $p_{b}^{g}\in R^{3}$ denotes the robot’s position in the global frame, $q_{b}^{g}\in R^{3}$ is its orientation (as a unit quaternion), and $v_{b}^{g}\in R^{3}$ is the velocity, respectively. The state is jointly estimated over a sliding window of K keyframes, determined by the motion-aware system and refined through nonlinear least-squares optimization, following prior work [4,40]. Under the assumption of zero-mean Gaussian noise, the joint cost function minimized during backend optimization is formulated as follows:

(15) $$\begin{array}{c} \chi^{*}=\arg\;\underset{\chi }{\min}\underset{Prior}{\underbrace{{\left\|r_{p}-H_{p}\chi \right\|}^{2}}}+\sum_{k=0}^{K-1}\omega_{IMU}{\left\|r_{k}^{IMU}(\chi )\right\|}^{2}+\\ \;\sum_{k=0}^{K-1}\sum_{l\in F_{k}}\omega_{feat}{\left\|r_{k,l}^{feat}(\chi )\right\|}^{2}+\sum_{(i,k)\in E}\omega_{event}{\left\|r_{i\to k}^{event}(\chi )\right\|}_{P_{ik}^{-1}}^{2}, \end{array}$$

where

- $r_{p}-H_{p}\chi$ is the prior residual term that encodes the marginalization prior, capturing historical information from states removed from the sliding window;
- $r_{k}^{IMU}\left(\chi \right)\;$ are the IMU pre-integration residuals, modeling motion between keyframes;
- $r_{k,l}^{feat}(\chi )$ represent reprojection errors from tracked visual landmarks (from both RGB and event-based features);
- $r_{i\to k}^{event}(\chi )$ are photometric alignment residuals between event mats, weighted by the inverse of the alignment Hessian $P_{ik}^{-1}$, reflecting the confidence of the direct event-based constraint. It should be noted that, in all equations, bold symbols denote vector quantities, whereas scalar parameters are represented in regular font.

In our hybrid odometry (MA-EVIO) system, each measurement modality—IMU, feature-based, and event-based—contributes complementary information to the state estimation process. However, the reliability and informativeness of each modality vary significantly depending on motion dynamics, sensor quality, and environmental conditions. To address this variability, we introduce motion-aware weighting parameters $\omega_{IMU}$, $\omega_{feat}$, and $\omega_{event}$ which adaptively adjust the contribution of each residual term in the joint optimization cost function.

#### 2.5.1. Prior Residual Term

The prior residual term represents the effect of previously estimated states that have been marginalized out of the current optimization window in the fixed-lag smoothing process. In the context of MAP estimation, it corresponds to the negative log likelihood of the prior distribution on the current system state χ, derived from past measurements that are no longer explicitly included in the sliding window.

During marginalization, both the Jacobian matrix $H_{P}$ and the residual vector $r_{p}$ are computed such that they implicitly encode the uncertainty from the eliminated variables. Specifically, the information matrix of the prior is approximately

(16) $$\Lambda_{prior}\approx H_{p}^{⊤}H_{p},$$

This makes the prior residual term equivalent to a Mahalanobis norm [41] with the corresponding information matrix already embedded:

(17) $${\left\|r_{p}-H_{p}\chi \right\|}^{2}={\left\|\chi -{\hat{\chi }}_{prior}\right\|}_{\Lambda_{prior}}^{2},$$

Importantly, no explicit weighting parameter is applied to this term. This is because the marginalization procedure inherently balances the contribution of the prior based on the observed data and the confidence (covariance) of the marginalized variables. Any artificial weighting (e.g., $\omega_{prior}$) could distort the optimizer’s behavior and potentially reduce numerical stability, especially in systems where accurate drift correction relies heavily on prior consistency.

#### 2.5.2. IMU Residuals Term

The IMU residuals encode the motion constraints between consecutive keyframes using pre-integrated inertial measurements, which are fused with other sensing modalities in the optimization backend. These residuals play a critical role in recovering the metric scale, estimating dynamic motion, and maintaining the temporal consistency of the trajectory. At the core of the formulation is the IMU pre-integration model, originally introduced in [42] and later refined in [43], which allows inertial measurements to be integrated over short intervals between keyframes, accounting for both bias and noise, without requiring re-integration at every iteration. Mathematically, the residual is structured as follows:

(18) $$r_{k}^{IMU}(\chi )=\left[\begin{array}{c} r_{p}\\ r_{q}\\ r_{v} \end{array}\right]\in R^{9},$$

where

- $r_{p}\in R^{3}\;$ is the position residual;
- $r_{q}\in R^{3}\;$ is the orientation residual (represented on the tangent space SO(3);
- $r_{v}\in R^{3}\;$ is the velocity residual.

These residuals are derived from the pre-integrated measurements $\alpha_{ij},\beta_{ij},\gamma_{ij}$, which represent changes in position, velocity, and orientation, respectively, over the interval $\left[t_{i},t_{j}\right]$ between two keyframes i and j:

(19) $$\begin{array}{c} \alpha_{ij}=\int_{t_{i}}^{t_{j}}\int_{t_{i}}^{s}R_{u}(a_{u}-b_{a})duds,\\ \beta_{ij}=\int_{t_{i}}^{t_{j}}R_{u}(a_{u}-b_{a})du,\\ \gamma_{ij}=\int_{t_{i}}^{t_{j}}\frac{1}{2}(\omega_{u}-b_{g})du, \end{array}$$

where

- $a_{u}$ is linear acceleration measured by the IMU at time u, in the IMU (body) frame.
- $b_{a}$ is a constant or slowly varying accelerometer bias, estimated as part of the state.
- $\omega_{u}$ is angular velocity measured by the gyroscope at time u, in the body frame.
- $b_{g}$ is a constant or slowly varying gyroscope bias, also estimated online.
- $R_{u}\in SO(3)$ is the rotation matrix representing the orientation of the IMU at time u and is used to rotate body-frame measurements to the global frame.

Each of these terms represents IMU-derived kinematic quantities over the interval $\left[t_{i},t_{j}\right]$. These pre-integrated terms are efficiently computed and held constant during optimization, with only the residual evaluated at each iteration. This avoids costly reintegration and improves computational efficiency.

The IMU provides high-rate motion constraints through pre-integration, which is essential for capturing short-term dynamics and maintaining scale, but integration errors and bias drift can accumulate, especially during low excitation or degraded sensing. To adaptively manage this, we assign a motion-aware weight $\omega_{IMU}$ to the inertial residuals. This weight is computed from the variance of gyroscope and accelerometer readings. When the system detects aggressive motion (e.g., high variance), $\omega_{IMU}$ is increased to emphasize to the reliable inertial information. Also, during static, or when the gyroscope or accelerometer saturates, $\omega_{IMU}$ is down-weighted to reduce the influence of potentially unreliable measurements. This strategy ensures that inertial constraints contribute meaningfully when informative, while mitigating drift in degenerate or saturated motion conditions.

#### 2.5.3. Feature-Based Reprojection Residuals

The feature reprojection residuals encode the geometric discrepancy between the observed 2D positions of features (in either RGB images or adaptive time surfaces from event data) and their predicted projections from estimated 3D landmarks. These constraints form the core of the visual part of the backend optimization, leveraging both spatial structure and temporal consistency. We define each residual as follows:

(20) $$r_{k,l}^{feat}\left(\chi \right)=z_{k,l}^{obs}-\pi (T_{c_{k}}^{w}.P_{l}),$$

where

- $z_{k,l}^{obs}\in R^{2}$ is the observed 2D feature location in the image (either RGB or event);
- $P_{l}\in R^{3}$ is the estimated 3D position of the landmark;
- $T_{c_{k}}^{w}\in R^{3}$ is the pose of the camera at keyframe k;
- π(⋅) denotes the perspective projection function with camera intrinsic parameters.

As discussed in Section 2.3, the hybrid tracking module extracts visual features from both RGB frames and adaptive time surfaces generated by the event stream. These features, once successfully extracted across multiple views, are triangulated to recover their 3D positions using inverse depth parameterization, which enhances numerical stability and is particularly effective in handling monocular scale ambiguity. The triangulation process is formulated as follows:

(21) $$P_{l}=\pi^{-1}(z_{i,l},d_{l}),$$

where $z_{i,l}$ denotes the first 2D observation of the feature, and $dl_{l}=1/\lambda$ represents its inverse depth. Features that meet promotion criteria are elevated to landmarks, and their 3D positions are jointly optimized via reprojection residuals across all observing keyframes, thereby contributing to the global motion estimation.

To ensure robust state estimation in indoor environments, where visual conditions may vary due to motion blur, low texture, or artificial lighting, we modulate the contribution of feature-based residuals using a motion-aware weight $\omega_{feat}$. This weight is dynamically adjusted based on two key metrics: the tracking success ratio, reflecting the proportion of inlier feature tracks over the sliding window, and local brightness consistency, capturing photometric stability around tracked features in RGB frames. When tracking degrades—e.g., due to rapid motion or lighting fluctuations—$\omega_{feat}$ is down-weighted to mitigate the influence of unreliable measurements. Conversely, under stable conditions with sufficient texture and consistent illumination, the weight is increased to fully exploit the geometric accuracy of spatial landmarks.

#### 2.5.4. Event Mat Alignment Residuals

The event-based direct alignment residuals, denoted as $r_{i\to k}^{event}(\chi )$, introduce dense pose constraints by leveraging the photometric information encoded in event mats. These residuals are designed to align the current event mat to a reference one via direct image alignment. In each residual block, the relative transformation between two poses $T_{W_{i}}^{W_{k}}\in SE(3)$ is estimated by minimizing the photometric error between the warped current event mat and the stored reference:

(22) $$E_{event}={\sum_{p\in Ω}\left\|I_{ref}\left(p\right)-I_{curr}(\pi \left(T.\pi^{-1}\left(p\right)\right))\right\|}^{2},$$

where

- $I_{ref}\left(p\right)$ and $I_{curr}$ denote the intensity values from the reference and current event mats, respectively;
- π and $\pi^{-1}$ are the camera projection and back-projection functions;
- T∈SE(3) is the estimated pose transformation;
- Ω is the set of pixels used for alignment.

The optimization is performed using IC-LK method [38], with the event mat alignment residuals optimized through the Ceres Solver framework [39]. This iterative refinement process leverages the residual structure to ensure accurate alignment between the event data and image features.

Each residual is further weighted by an information matrix $P_{ik}^{-1}$ derived from the Gauss–Newton approximation [44] of the Hessian during alignment [34]:

(23) $$r_{i\to k}^{event}\left(\chi \right)=P_{ik}^{-\frac{1}{2}}.\log\left(T_{meas}^{-1}.T\left(\chi \right)\right)$$

where

- log(.) maps from SE(3) to its tangent space;
- $T_{meas}$ is the relative pose estimated from direct alignment of event mats;
- χ denotes the full system state, including the relevant poses.

These residuals offer dense and drift-resistant constraints in high-frequency regimes where traditional features become sparse or unreliable. They are particularly effective in challenging motion scenarios (e.g., rapid rotations or low-texture scenes) and contribute significantly to enhancing the robustness of the VIO system. In our optimization pipeline, the event-based residuals are modulated by a dynamic weight parameter $\omega_{event}$, which reflects the confidence in the direct alignment between event mats. This weight is adaptively adjusted based on three main factors: residual convergence, spatial distribution of events, and motion intensity. High alignment quality—characterized by low residuals and dense, uniformly distributed events—leads to an increased $\omega_{event}$, reinforcing the influence of reliable direct constraints. This mechanism becomes particularly beneficial in feature-sparse indoor environments, where RGB or corner-based features are unreliable. In such cases, the system assigns greater weight to event-based alignment, leveraging its robustness to low texture and high-speed motion. Conversely, in the presence of features in static or slow motion, sparse event coverage, or poor convergence, $\omega_{event}$ is down-weighted to mitigate the influence of potentially noisy constraints, thus preserving the stability and accuracy of the hybrid optimization. It is important to emphasize that the system maintains a single set of state variables shared across both constraint types, with synchronization performed prior to each optimization cycle.

## 3. Dataset

To rigorously evaluate the performance, accuracy, and robustness of our MA-EVIO system, we conduct experiments using two publicly available and widely adopted datasets: the Event-Camera Dataset and Simulator (DAVIS240c) [28] and the VECtor Benchmark [29]. According to the dataset documentation [28,29], both datasets ensure hardware-level temporal synchronization across sensors and provide full intrinsic and extrinsic calibration parameters, with inter-sensor clock drift either negligible or explicitly compensated by the data provider. Building on these guarantees, we also conducted an independent software-level verification of temporal consistency by monitoring timestamp alignment across the camera, event, and IMU streams, and discarding any samples that exceeded a predefined synchronization tolerance. This verification step ensured that only temporally consistent multi-sensor measurements were used in our experiments and further strengthened the reproducibility and reliability of the reported results. Together, they enable thorough benchmarking across diverse environments, sensor configurations, and motion dynamics, making them ideal for assessing the real-world applicability of EVIO pipelines.

**Figure 5:**
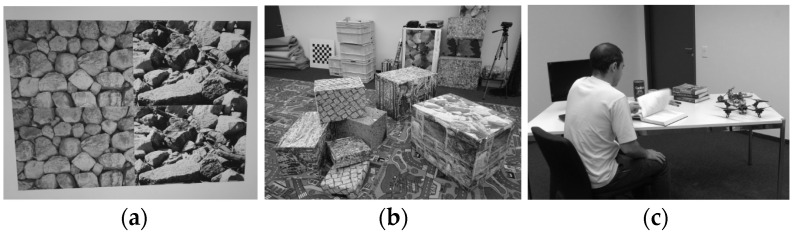
Different scenes of the dataset used in the event camera SOTA comparison with our proposed technique, (**a**) wall poster, (**b**) boxes, (**c**) dynamic. Images are modified from [28].

The DAVIS240c dataset [28] is one of the earliest and most widely used benchmarks for event-based pose estimation, visual odometry, and simultaneous localization and mapping (SLAM). It features data recorded with a DAVIS240C sensor, which outputs both asynchronous events and grayscale images at high temporal resolution, as well as IMU measurements from the integrated inertial sensor. The dataset includes motions ranging from slow and smooth to rapid and aggressive, including high-speed rotations and fast translations, making it well-suited to evaluate both frame-based and event-driven tracking systems. Additionally, the dataset includes a simulator, allowing for synthetic data generation under controlled conditions for algorithm development and ablation studies. Figure 5 provides sample images from selected sequences in the DAVIS240c dataset.

**Figure 6:**
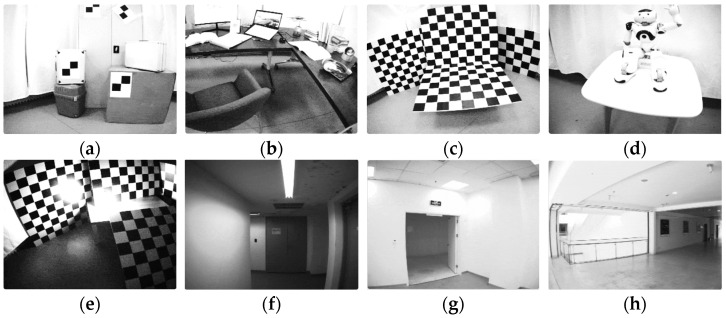
Different scenes of the dataset used in the event camera SOTA comparison with our proposed technique, (**a**) corner, (**b**) desk, (**c**) mountain, (**d**) robot, (**e**) HDR, (**f**) corridor, (**g**) units, and (**h**) school. Image are modified from [29].

The VECtor dataset [29] is a more recent and comprehensive benchmark specifically tailored for multi-sensor EVIO evaluation. It includes a rich variety of sequences captured using a multi-modal rig equipped with stereo event cameras (Prophesee Gen3), stereo standard RGB cameras (Grasshopper3), an industrial-grade IMU, and LiDAR for large scale sequences or RGB-D depth camera for small scale sequences. Ground truth trajectories are obtained using a high-precision motion capture system, which tracks the position and orientation of objects using infrared cameras and reflective markers. Additional ground truth is generated using the Iterative Closest Point (ICP) method for alignment in cases where motion capture is unavailable. VECtor covers a wide range of platforms—including hand-held and wheeled and aerial robots in structured indoor spaces. It also includes complex motion profiles such as rapid camera shake, sudden accelerations, and large rotations. Importantly, VECtor supports rigorous evaluation with multi-sensor time synchronization, enabling fair comparisons between event-only, visual–inertial, and LiDAR-based odometry systems. Figure 6 provides sample images from selected sequences of the VECtor dataset.

## 4. Results

We present the evaluation results of the proposed MA-EVIO framework using the DAVIS240C and VECtor datasets (Section 3). The system is implemented in C++ under Ubuntu 20.04 using ROS Noetic (ROS 1 distribution-version 1.17.0). All sequences are processed in real time. Experiments were conducted on a Dell Precision 5820 workstation [45] equipped with an NVIDIA T1000 (8 GB) GPU [46] and an Intel^®^ Xeon^®^ W-2235 CPU [47]. The frontend runs at 16–33 ms per frame, while the backend optimization requires 20–40 ms per iteration and is executed asynchronously. The motion-aware module introduces less than 0.5 ms overhead per cycle. The system maintains stable real-time performance more than 15 Hz. In terms of memory consumption, the framework requires 4–7 MB during normal operation, with a peak usage of 15–18 MB when loop closure is enabled. These results indicate that MA-EVIO is suitable for deployment on resource-constrained onboard platforms.

The first set of experiments is conducted on the DAVIS240c dataset, where we compare our approach against two baseline methods to demonstrate its effectiveness. To further examine the model’s generalizability, we perform benchmarking on the VECtor dataset against leading approaches in the field. Detailed analyses of these evaluations are presented in the following subsections.

The results of our evaluation on the DAVIS240C dataset [28] are summarized in Table 1 and Table 2. Table 1 presents trajectory accuracy values reported by state-of-the-art (SOTA) methods evaluated under a consistent protocol, in which each estimated trajectory is aligned with the ground truth over the initial 0–5 s interval. The trajectory accuracy results are expressed as percentages; for example, an error of 0.15% corresponds to a deviation of 0.15 m over a 100 m trajectory.

Table 2 reports the outcomes of our quantitative assessment using the evo package [48], which provides standardized metrics for the accuracy and consistency of trajectory estimation. The listed measures include maximum (Max), mean, median, minimum (Min), and root mean square error (RMSE), offering a comprehensive representation of both accuracy and consistency in trajectory estimation. For comparison, we benchmark our MA-EVIO approach against other techniques with publicly available full trajectories [14,49].

**Table 1:** Comparative accuracy evaluation of the proposed MA-EVIO model against EIO and EVIO methods on the DAVIS240c dataset [28].

Methods	boxes_ translation	hdr_ boxes	boxes _6dof	dynamic_ translation	dynamic _6dof	poster_ translation	hdr_ poster	poster_ 6dof	Average of Results
CVPR17 EIO [50]	2.69	1.23	3.61	1.9	4.07	0.94	2.63	3.56	2.58
BMVC17 EIO [51]	0.57	0.92	0.69	0.47	0.54	0.89	0.59	0.82	0.69
U-SLAM EIO [14]	0.76	0.67	0.44	0.59	0.38	0.15	0.49	0.3	0.47
U-SLAM EVIO [14]	0.27	0.37	0.30	**0.18**	**0.19**	**0.12**	0.31	0.28	0.25
3DV19 EIO [52]	2.55	1.75	2.03	1.32	0.52	1.34	0.57	1.50	1.45
EKLT-VIO [18]	0.48	0.46	0.84	0.4	0.79	0.35	0.65	0.35	0.54
IROS22 EIO [53]	1.0	1.8	1.5	0.9	1.5	1.9	2.8	1.2	1.58
Mono-EIO [49]	0.34	0.40	0.61	0.26	0.43	0.4	0.4	0.26	0.39
PL-EVIO [17]	**0.06**	**0.10**	0.21	0.24	0.48	0.54	0.12	**0.14**	0.24
Event AC SLAM [54]	0.28	0.27	0.29	0.24	0.29	0.13	0.3	0.21	0.25
Tang et al. [55]	0.36	0.31	0.32	0.59	0.49	0.23	0.18	0.31	0.35
EVI-SAM [27]	0.11	0.13	**0.16**	0.30	0.27	0.34	**0.15**	0.24	0.21
MA-EVIO (Our Method)	0.17	0.23	0.23	0.21	0.22	**0.12**	0.22	0.17	**0.19**

The symbols E, V, and I refer to the use of event data, visual image, and IMU measurements, respectively. The best performing method in every sequence is highlighted in bold.

The evaluation on the VECtor dataset [29] is presented in Table 3, using the same evaluation criteria [56] to ensure consistency across datasets. The reported metrics include the mean position error (MPE, %) and the mean rotation error (MRE, deg/m). The evaluation aligns the complete ground-truth trajectory with the estimated poses, ensuring consistent computation of both translation and rotation errors. By incorporating the original values from the respective publications, Table 2 and Table 3 provide a fair and direct comparison of SOTA methods under uniform evaluation conditions.

**Table 2:** Performance comparison of our model with baseline techniques on DAVIS240C dataset (errors in cm).

Sequence	Metric	U-SLAM- EVIO [14]	IROS22- EIO [49]	MA-EVIO (Our Method)
boxes_6dof	Max	58.84	200.12	**46.04**
	Mean	25.69	75.94	**17.36**
	Median	24.31	61.72	**15.91**
	Min	12.60	41.99	**06.65**
	RMSE	27.04	84.32	**18.47**
boxes_ translation	Max	40.21	94.45	**30.23**
	Mean	21.08	42.46	**14.96**
	Median	19.03	38.45	**14.04**
	Min	09.87	19.05	**06.84**
	RMSE	21.95	44.75	**15.52**
dynamic_ 6dof	Max	29.69	114.16	**23.66**
	Mean	14.35	28.60	**11.52**
	Median	13.73	24.56	**10.70**
	Min	06.03	10.67	**02.66**
	RMSE	14.62	31.79	**12.35**
dynamic_ translation	Max	**25.47**	40.25	45.05
	Mean	09.29	12.78	**08.12**
	Median	09.04	11.09	**07.25**
	Min	05.80	04.20	**02.96**
	RMSE	09.41	13.96	**08.96**
hdr_boxes	Max	90.23	109.80	**44.87**
	Mean	22.85	29.91	**16.36**
	Median	20.77	23.51	**14.94**
	Min	13.63	11.47	**05.34**
	RMSE	24.49	33.20	**17.52**
hdr_poster	Max	88.31	78.33	**32.67**
	Mean	**15.54**	22.59	18.38
	Median	**13.46**	21.73	18.45
	Min	06.88	06.39	**03.29**
	RMSE	**17.28**	23.47	19.15
poster_6dof	Max	63.77	225.99	**42.31**
	Mean	33.36	30.32	**16.28**
	Median	32.69	21.65	**15.07**
	Min	21.12	5.04	**04.31**
	RMSE	33.63	39.02	**17.29**
poster_translation	Max	**15.79**	119.46	20.20
	Mean	**06.30**	39.27	08.51
	Median	**05.57**	35.32	08.10
	Min	**01.52**	18.16	01.94
	RMSE	**06.91**	41.74	08.98

The notations E, V, and I stand for the use of event, visual image, and IMU, respectively. The best performance result for each method in every sequence is highlighted in bold.

**Table 3:** Accuracy comparison between the proposed MA-EVIO method and existing approaches on the VECtor dataset [29].

Sequence	ORB-SLAM3 [57] (SVIO)	VINS-Fusion [58] (SVIO)	EVO [21]	ESVO [23]	U-SLAM [14] (EVIO)	PL-EVIO [17]	EVI-SAM [27]	MA-EVIO (Our Method)
	MPE/MRE	MPE/MRE	MPE/MRE	MPE/MRE	MPE/MRE	MPE/MRE	MPE/MRE	MPE/MRE
corner-slow	**1.49**/14.28	1.61/**14.06**	4.33/15.52	4.83/20.98	4.83/14.42	2.10/14.21	2.50/14.82	1.98/14.46
robot-normal	0.73/1.18	**0.58**/1.18	3.25/2.00	failed	1.18/1.11	0.68/1.25	0.67/**0.85**	0.65/1.05
robot-fast	0.71/0.70	failed	failed	failed	1.65/0.56	0.17/0.74	0.22/**0.41**	**0.16**/0.67
desk-normal	**0.46**/0.41	0.47/0.36	failed	failed	2.24/0.56	3.66/0.45	1.45/**0.28**	1.59/0.30
desk-fast	0.31/0.41	0.32/0.33	failed	failed	1.08/0.38	**0.14**/0.48	0.18/**0.38**	0.20/0.46
sofa-normal	0.15/0.41	**0.13**/0.40	failed	1.77/0.60	5.74/0.39	0.19/0.46	0.19/**0.20**	0.19/0.33
sofa-fast	0.21/0.43	0.57/0.34	failed	failed	2.54/0.36	**0.17**/0.47	0.98/**0.31**	0.47/0.32
mountain- normal	**0.35**/1.00	4.05/1.05	failed	failed	3.64/1.06	4.32/0.76	1.39/**0.65**	0.78/0.96
mountain-fast	2.11/0.64	failed	failed	failed	4.13/0.62	**0.13**/0.56	0.38/**0.30**	0.32/0.48
hdr-normal	**0.64**/1.20	1.27/1.10	failed	failed	5.69/1.65	4.02/1.52	5.74/**0.87**	4.63/1.36
hdr-fast	0.22/0.45	0.30/0.34	failed	failed	2.61/0.34	**0.20**/0.50	0.67/**0.26**	0.73/0.47
corridors-dolly	**1.03**/1.37	1.88/1.37	failed	failed	failed	1.58/1.37	1.58/1.38	1.81/**0.30**
corridors-walk	1.32/1.31	**0.50**/1.31	failed	failed	failed	0.92/1.31	1.27/1.35	0.63/**0.12**
school-dolly	0.73/1.02	**1.42**/1.06	failed	10.87/1.08	failed	2.47/0.97	1.53/0.89	1.99/**0.16**
school-scooter	0.70/0.49	**0.52**/0.61	failed	9.21/0.63	6.40/0.61	1.30/0.54	1.46/0.53	1.96/**0.13**
units-dolly	7.64/0.41	4.39/0.42	failed	failed	failed	5.84/0.44	**0.59**/0.35	1.02/**0.08**
units-scooter	6.22/0.22	4.92/0.24	failed	failed	failed	5.00/0.42	**0.83**/0.38	1.19/**0.10**
Average	1.47/1.53	1.53/1.61	3.79/8.76	6.67/5.82	3.48/1.84	1.93/1.56	1.27/1.42	**1.19/1.28**

The symbols E, F, S, and I denote the use of event data, frame images, stereo, and IMU measurements, respectively. The best performance result for each method in every sequence is highlighted in bold. Rows marked as “failed” were excluded from the average calculation.

## 5. Discussion

The effectiveness of the proposed MA-EVIO system is evaluated on two publicly available benchmarks. To ensure adequate comparison, trajectory estimates are aligned with ground truth using the evo Python package [48], which follows a standardized evaluation protocol.

Table 1 presents a comprehensive comparative evaluation of the proposed MA-EVIO framework against a range of SOTA EIO and EVIO methods on the DAVIS240C dataset. All methods follow a consistent evaluation protocol in which estimated trajectories are aligned with the ground truth over the initial 0–5 s interval, with accuracy expressed as a percentage of trajectory deviation. The results highlight the competitive performance of MA-EVIO across diverse scenarios, including dynamic motion, HDR conditions, and feature-sparse environments. On average, our method achieves an error rate of 0.19%, outperforming several purely event-based approaches, such as IROS22 EIO [53] and 3DV19-EIO [45], and approaching the performance of advanced hybrid systems like EVI-SAM [23]. Notably, MA-EVIO maintains consistently low errors across all sequences, with particularly strong results in challenging HDR and dynamic motion settings, underscoring the benefits of our motion-aware optimization and hybrid event–frame–IMU integration. These findings demonstrate that the proposed framework achieves a favorable balance between accuracy and robustness, validating its effectiveness for reliable visual–inertial odometry in complex indoor scenarios.

The results in Table 2 clearly demonstrate the advantage of our MA-EVIO framework over both U-SLAM [14] and IROS22-EIO [42]. Across most tested sequences, our method consistently achieves a lower mean, median, and RMSE, highlighting its robustness in the diverse indoor scenarios. For instance, in the boxes 6DoF sequence, MA-EVIO reduces the RMSE to 18.47 cm, compared to 27.04 cm with U-SLAM and 84.32 cm with IROS22-EIO. Similarly, in the challenging dynamic translation sequence, our method achieves an RMSE of 12.35 cm, a substantial improvement over both baselines.

A particularly notable improvement is observed in high-dynamic-range (HDR) environments (e.g., hdr boxes and hdr poster sequences), where traditional frame-based methods often struggle due to photometric distortions. Here, the event-based alignment in our system proves highly effective, reducing RMSE from 33.20 cm with IROS22-EIO to 17.52 cm. Likewise, in the box translation sequence, MA-EVIO achieves an RMSE of 15.52 cm, which is significantly lower than 21.95 cm with U-SLAM and 44.75 cm with IROS22-EIO, highlighting its ability to maintain accurate translation estimates even under complex motion.

**Figure 7:**
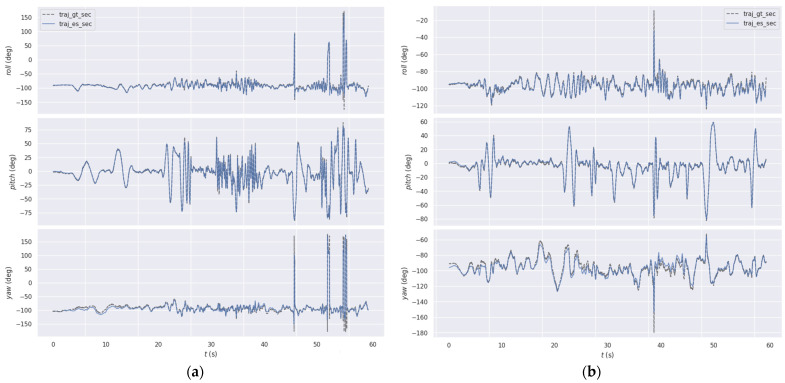
Comparing the estimated trajectory in terms of rotation produced by our MA-EVIO with the ground truth trajectory in two sequences of DAVIS240c dataset [28]: (**a**) poster_6dof; (**b**) hdr_poster.

In Figure 7, we compare the estimated rotational trajectories from MA-EVIO with the ground truth for two representative sequences of the DAVIS240c dataset [28]. Across all cases, the estimated roll, pitch, and yaw show strong alignment with the ground truth, indicating accurate orientation tracking under diverse indoor conditions. The results demonstrate that the system maintained stability even during rapid and dynamic motions, accurately capturing complex six-degree-of-freedom movements with minimal drift. Furthermore, it showed strong robustness under challenging illumination conditions, where traditional frame-based methods typically degrade, with event-based measurements ensuring reliable performance. Collectively, these findings highlight the effectiveness of MA-EVIO in maintaining accurate rotational estimates across a wide range of motion dynamics and lighting scenarios.

To further assess the generalizability and robustness of the proposed MA-EVIO system, we evaluate its performance on the VECtor benchmark dataset, which contains a rich variety of both small-scale and large-scale indoor sequences characterized by dynamic motion, HDR lighting, and significant texture variations. Table 3 presents a comprehensive comparison between MA-EVIO and several SOTA SLAM and EVIO approaches. The evaluation is reported in terms of mean position error (MPE) and mean rotation error (MRE) across a diverse set of sequences. MA-EVIO consistently demonstrates superior performance, particularly in challenging high-speed and low-texture scenarios where many traditional and event-based methods fail or degrade significantly. For example, in the robot-fast sequence, MA-EVIO achieves the lowest MPE of 0.16 m, outperforming all baselines including EVI-SAM (0.22 m), while maintaining a competitive rotational error of 0.67°, highlighting its effective motion-aware integration and robust optimization framework.

MA-EVIO also proves to be resilient in high-dynamic-range and cluttered environments. In the hdr-normal sequence, although many methods either fail or exhibit high errors, MA-EVIO delivers competitive results with an MPE/MRE of 4.63 m/1.36°. While PL-EVIO achieves a slightly lower positional error (4.02 m), our method demonstrates superior rotational stability, with an error significantly lower than U-SLAM (1.65°) and PL-EVIO (1.52°). Even in large-scale scenes like units-scooter, which challenge most methods, MA-EVIO maintains a strong performance at 1.19 m/0.10°, vastly outperforming VINS-Fusion (4.92 m/0.24°) and ORB-SLAM3 (6.22 m/0.22°). Notably, the system shows stability in both slow and fast motion conditions—such as in desk-fast and sofa-fast—while maintaining consistently low rotational drift. These findings collectively demonstrate that MA-EVIO is not only robust across lighting and motion variations but also scalable to larger spatial domains, making it well-suited for a broad range of real-world indoor navigation and localization applications.

To investigate the dominant factors contributing to trajectory drift, we check error traceability analysis on representative sequences. By analyzing the residual distribution, we observe that peak errors in the MA-EVIO system are strongly correlated with specific environmental triggers. In HDR scenarios, such as the hdr-normal sequence, error spikes align primarily with moments of lighting transition during motion, where the event generation rate fluctuates significantly, temporarily reducing the effective constraint from visual features. Conversely, in highly dynamic sequences such as robot-fast, the primary source of error shifts to IMU bias integration during periods of extremely rapid rotation, where the duration of reliable visual feature tracking is minimized. A quantitative breakdown of error contributions shows that, while the visual–inertial alignment module accounts for the largest proportion of drift in texture-sparse areas, the motion-aware optimization successfully mitigates most large-scale deviations, preventing local errors from accumulating into significant global drift. This analysis confirms that the proposed hybrid system effectively compensates for individual sensor limitations, although extreme and abrupt lighting transitions remain a key area for further improvement.

Despite the notable accuracy of MA-EVIO across both DAVIS240C and VECtor datasets, several limitations remain that offer opportunities for future enhancement. One observed limitation is the system’s sensitivity to large-scale sequences involving extended motion trajectories and limited loop closure opportunities, such as in the units-dolly and units-scooter scenes from the VECtor dataset. Although MA-EVIO outperforms many baselines in these cases, the performance gap compared to smaller-scale environments suggests that drift accumulates over long distances due to limited global correction mechanisms. Since the current system relies primarily on local tracking through event, frame, and inertial data, it lacks robust place recognition or mapping components that can correct accumulated drift over time. Furthermore, while MA-EVIO performs well with high-fidelity inertial data, its reliance on precise time synchronization and accurate extrinsic calibration between sensors can make it sensitive to small calibration errors, particularly in real-world deployments where factory-grade sensor alignment may not be guaranteed.

To address these limitations, future work can focus on enhancing the spatial observability of the system by integrating depth information from LiDAR or RGB-D sensors. The inclusion of LiDAR data would provide complementary structural cues, especially beneficial in large-scale or geometrically repetitive environments where visual features are insufficient or unreliable. A hybrid fusion pipeline that leverages dense LiDAR point clouds, sparse event features, and inertial cues could mitigate drift in open spaces and improve loop closure capabilities. Furthermore, incorporating more adaptive sensor selection or dynamic weighting strategies—based on environmental characteristics—could allow MA-EVIO to generalize better across diverse scenes. Finally, extending the system for real-time deployment on edge devices with hardware-aware optimizations remains an important direction for future development.

## 6. Conclusions

In this study, we proposed MA-EVIO, a motion-aware event-based visual–inertial odometry system that adaptively fused asynchronous event data, frame-based imagery, and inertial measurements for robust pose tracking. The system employed a hybrid tracking strategy that combined sparse feature matching with direct photometric alignment, striking a balance between robustness and precision. A key contribution was the motion-aware keyframe selection mechanism, which dynamically adjusted tracking parameters based on real-time motion classification and feature quality. This design enabled adaptive sensor fusion, prioritizing event data and direct methods during fast motion while leveraging RGB frames and feature-based tracking under slower motion. Quantitative results confirmed the effectiveness of MA-EVIO; for example, on the DAVIS240C dataset, it achieved an average trajectory error of 0.19%, improving upon leading EVIO baselines such as EVI-SAM (0.21%), U-SLAM-EVIO (0.25%), and IROS22-EIO (1.58%). On the VECtor dataset, MA-EVIO achieved the lowest mean position and rotation errors in some sequences—such as 0.16/0.67° in the robot-fast sequence and 0.63/0.12° in corridors-walk—demonstrating superior robustness under high-speed and HDR conditions. Consequently, MA-EVIO provided reliable localization in GNSS-denied indoor environments, advancing the SOTA techniques in EVIO.

## Data Availability

The data presented in this study are available upon request from the corresponding author. The system is currently under active development and is being prepared for the next phase of research.
